# Supplementary material for: Pt Nanoparticles with High Oxidase-Like Activity and Reusability for Detection of Ascorbic Acid
Source: Nanomaterials (Basel). 2020 May 26;10(6):1015. doi: 10.3390/nano10061015 (PMC7352317; doi:10.3390/nano10061015)
Supplement: Supplementary file 1 [file nanomaterials-10-01015-s001.pdf]

Supplementary Information

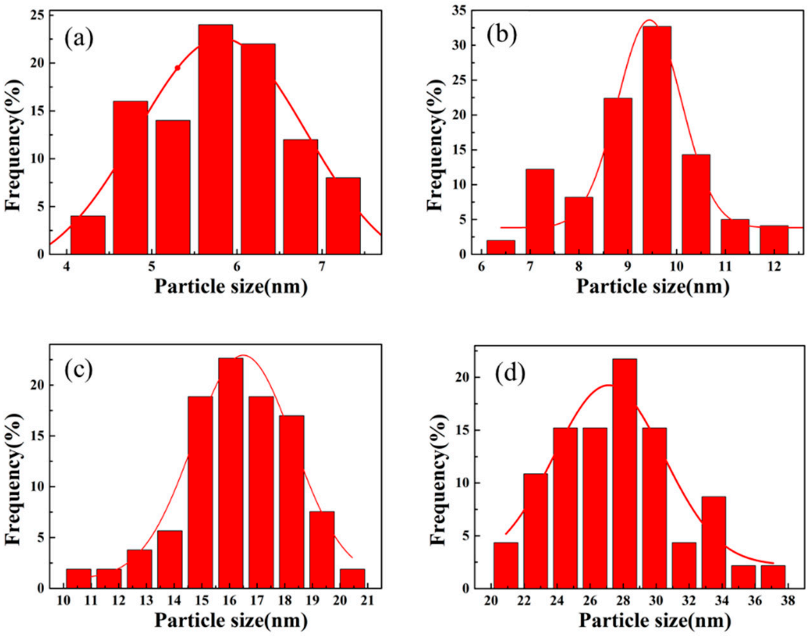

Figure S1 Size distributions of (a) Pt-0 HCl, (b) Pt-3.7 HCl, (c) Pt-9.25 HCl and (d) Pt-2 PVP.

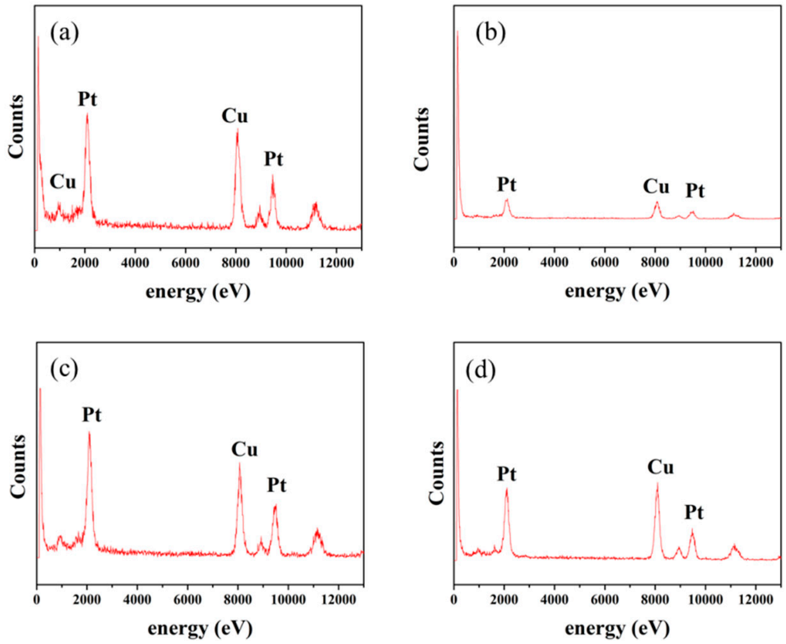

Figure S2 EDS elemental analysis of
(a) Pt-0 HCl, (b) Pt-3.7 HCl, (c) Pt-9.25 HCl and (d) Pt-2 PVP.

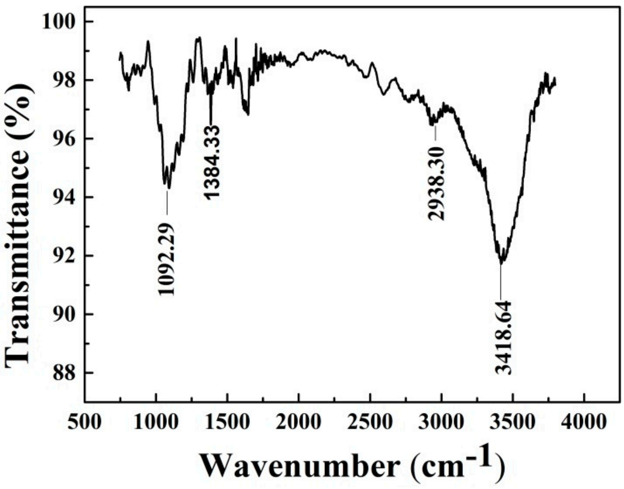

Fig. S3 FTIR spectra of Pt NPs.

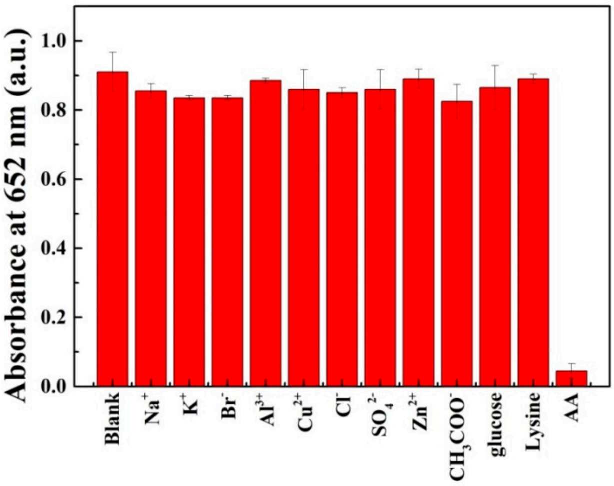

Figure S4 Interference of different interfering substances in the AA detection， all interfering substances were at a concentration of 25 μM.

Table S1 Comparative table of steady-state kinetic parameter for Pt NPs and other materials

Catalyst	Substrate	Km (mM)	Ref.
Hg2+/citrate-AgNPs		0.23	[1]
CS-SeNPs		0.852	[2]
$NiCo_2O_4$ MS		0.127	[3]
Lysozyme-Pt NPs	TMB	0.63	[4]
Ni/Pd hollow NPs		0.11	[5]
Cu-Ag/rGO		0.634	[6]
Fe_3O_4@C		0.38	[7]
Pt NPs		0.102	This work

References.

1. Wang, G.-L.; Xu, X.-F.; Cao, L.-H.; He, C.-H.; Li, Z.-J.; Zhang, C. Mercury(II)-stimulated oxidase mimetic activity of silver nanoparticles as a sensitive and selective mercury(II) sensor. Rsc Advances 2014, 4, 5867-5872, doi:10.1039/c3ra45226c.
2. Cao, H.; Xiao, J.; Liu, H. Enhanced oxidase-like activity of selenium nanoparticles stabilized by chitosan and application in a facile colorimetric assay for mercury (II). Biochemical Engineering Journal 2019, 152, doi:10.1016/j.bej.2019.107384.
3. Su, L.; Dong, W.; Wu, C.; Gong, Y.; Zhang, Y.; Li, L.; Mao, G.; Feng, S. The peroxidase and oxidase-like activity of NiCo2O4 mesoporous spheres: Mechanistic understanding and colorimetric biosensing. Analytica Chimica Acta 2017, 951, 124-132, doi:10.1016/j.aca.2016.11.035.
4. Yu, C.-J.; Chen, T.-H.; Jiang, J.-Y.; Tseng, W.-L. Lysozyme-directed synthesis of platinum nanoclusters as a mimic oxidase. Nanoscale 2014, 6, 9618-9624, doi:10.1039/c3nr06896j.
5. Wang, Q.; Zhang, L.; Shang, C.; Zhang, Z.; Dong, S. Triple-enzyme mimetic activity of nickel-palladium hollow nanoparticles and their application in colorimetric biosensing of glucose. Chemical Communications 2016, 52, 5410-5413, doi:10.1039/c6cc00194g.
6. Darabdhara, G.; Sharma, B.; Das, M.R.; Boukherroub, R.; Szunerits, S. Cu-Ag bimetallic nanoparticles on reduced graphene oxide nanosheets as peroxidase mimic for glucose and ascorbic acid detection. Sensors and Actuators B-Chemical 2017, 238, 842-851, doi:10.1016/j.snb.2016.07.106.
7. An, Q.; Sun, C.; Li, D.; Xu, K.; Guo, J.; Wang, C. Peroxidase-Like Activity of Fe_3O_4@Carbon Nanoparticles Enhances Ascorbic Acid-Induced Oxidative Stress and Selective Damage to PC-3

Prostate Cancer Cells. Acs Applied Materials & Interfaces 2013, 5, 13248-13257, doi:10.1021/am4042367.
